# Impact of Age on Outcomes in Hospitalized Patients with Hereditary Hemorrhagic Telangiectasia

**DOI:** 10.1155/2018/4798425

**Published:** 2018-02-11

**Authors:** Vivek N. Iyer, Waleed Brinjikji, Dinesh Apala, Bibek S. Pannu, Aditya Kotecha, Michael D. Leise, Patrick S. Kamath, Sanjay Misra, Giuseppe Lanzino, Michael J. Krowka, Christopher P. Wood, Karen L. Swanson

**Affiliations:** ^1^Division of Pulmonary and Critical Care Medicine, Mayo Clinic, Rochester, MN, USA; ^2^Department of Radiology, Mayo Clinic, Rochester, MN, USA; ^3^Division of Gastroenterology and Hepatology, Mayo Clinic, Rochester, MN, USA; ^4^Department of Neurosurgery, Mayo Clinic, Rochester, MN, USA; ^5^Division of Pulmonary and Critical Care Medicine, Mayo Clinic, Scottsdale, AZ, USA

## Abstract

**Background:**

There is little published literature regarding the impact of age on outcomes amongst hospitalized HHT (hereditary hemorrhagic telangiectasia) patients.

**Methods:**

The Nationwide Inpatient Sample (NIS) was used to obtain data on all hospital discharges occurring in HHT patients from 2000 to 2012. The association between admission age and HHT-related complications and outcomes were studied.

**Results:**

10293 hospitalizations in HHT patients from 2000 to 2012 were included. Patients > 50 accounted for 77% of all admissions with 30% of admissions occurring in the 51–65 age group. Bleeding related complications were the most frequent (62.7%, *n* = 6455 hospitalizations), followed by cardiovascular (41%, *n* = 4216), neurological (12.4%, *n* = 1276), and hepatobiliary (6.4%, *n* = 660) complications. Patients older than 50 accounted for 83% of bleeding events, 90% of cardiovascular events, 58% of neurologic events, and 81% of hepatobiliary events. The vast majority (83%) of medical and surgical procedures were performed in those older than 50 years of age. Older patients also experienced higher rates of death.

**Conclusion:**

Aging has significant adverse impacts on rates of hospitalization, complications, and outcomes amongst HHT patients in the United States. Except for neurologic complications, the vast majority of this disease burden is borne by patients older than 50 years.

## 1. Introduction

Hereditary Hemorrhagic Telangiectasia (HHT) is a rare autosomal dominant vascular disorder characterized by the presence of large and small arteriovenous malformations in a number of vascular beds. HHT has an estimated prevalence of 1 in 5,000 to 1 in 10,000 individuals and is diagnosed using the Curacao criteria [1]. HHT has a unique phenotype in each affected individual even within the same family and this phenotype typically evolves over a patient's lifetime. This impact of aging on HHT-related outcomes was studied in 324 HHT patients by Plauchu et al. who showed that older HHT patients had a higher prevalence of severe gastrointestinal (GI) bleeding as well as liver involvement whereas lung and central nervous system (CNS) involvement were more frequent in younger individuals [2]. European studies have also looked at morbidity and mortality amongst older versus younger HHT patients and have compared them to the general population [3–6]. These studies have generally involved smaller numbers of HHT subjects with insufficient data regarding rates of specific complications amongst different age groups. Using a large nationwide database of hospitalized patients, we have previously reported on the high rates of bleeding related complications and outcomes amongst hospitalized HHT patients treated across the United States [7]. In the present study, we use this same database to explore the relationship between age and outcomes amongst a large cohort of hospitalized HHT patients across the United States.

## 2. Materials and Methods

### 2.1. Patient Population

We obtained a representative sample of hospitalized HHT patients in the United States by using the Nationwide Inpatient Sample (NIS) database. The Nationwide Inpatient Sample (NIS) is a large administrative database that contains a yearly record of* 20% of all* discharges (randomly selected) from* all nonfederal hospitals* in the United States. Data for roughly 7-8 million hospital discharges are recorded in the NIS annually. Each individual hospitalization is assigned 1 primary discharge diagnosis code and up to 23 distinct secondary diagnoses codes. Similarly procedural codes for up to 14 distinct types of procedures performed during any individual hospitalization are also recorded [8]. Using NIS data from 2000–2012, we identified all patients with a discharge diagnosis (primary or secondary) of HHT (ICD-9 diagnosis code of 448.0). Detailed information on the NIS is available at https://www.hcup-us.ahrq.gov/databases.jsp.

### 2.2. Demographics and Baseline Patient Characteristics

We recorded the following baseline patient characteristics: age, gender, and race. Age was categorized into four groups: <18, 18–50, 51–65, and >65 and race was categorized as White, Black, Hispanic, and Asian exactly as recorded in the NIS database. Baseline comorbidities studies included smoking status, presence of hypertension, presence of diabetes mellitus, and Charlson comorbidities index (CCI). The Charlson comorbidity index is a validated prognostic index for administrative use that has been shown to be predictive of 10-year mortality [9].

### 2.3. Complications and Outcomes Studied

Complications were divided into hemorrhagic, cardiopulmonary, neurologic, and hepatobiliary related. Hemorrhagic complications included anemia, gastrointestinal bleeding, epistaxis, hemoptysis, and hematemesis. Cardiopulmonary complications included congestive heart failure (CHF), acute myocardial infarction (MI), coronary artery disease, atrial fibrillation, ventricular fibrillation/cardiopulmonary arrest, deep venous thrombosis (DVT), pulmonary embolism, pulmonary hypertension, and pulmonary arteriovenous malformation (PAVM). Neurologic complications studied included acute ischemic stroke, transient ischemic attack (TIA), intracranial hemorrhage (ICH), subarachnoid hemorrhage (SAH), seizure, headache, cerebral abscess, brain arteriovenous malformation (BAVM), and spinal vascular malformation. Hepatobiliary complications included cirrhosis, portal hypertension, acute and chronic liver failure, jaundice, and biliary complications. In addition to diagnoses, we studied procedural utilization including cardiac catheterization, cardiopulmonary resuscitation, hemodialysis, blood transfusion, upper and lower gastrointestinal endoscopy, liver transplantation, inferior vena cava (IVC) filter placement, and epistaxis management including nasal packing, endovascular therapy, and surgical ligation.

Outcomes studied included in-hospital mortality, discharge location (home, short term care facility versus a long term care facility) hospital length of stay (LOS), rates of iatrogenic complications, and hospital billing data. All complications and outcomes were compared according to the 4 different age groups as noted above.

### 2.4. Statistical Analysis

All statistical analysis was performed using the SAS-based statistical software package JMP13.0 (https://www.jmp.com, Cary, NC). All categorical variables were compared using the chi-squared test. All continuous variables were studied using the Student's *t*-test. For the purposes of statistical analysis NIS discharge weights were not applied.

## 3. Results

### 3.1. Patient Characteristics

Baseline patient characteristics are shown in Table 1. A total of 10293 hospitalizations in HHT patients occurred over the study period with a mean age of 60.7 ± 19.1 years. 77% of HHT hospitalizations occurred in patients > 50 years of age and 60% of hospitalizations occurred in females. The majority of admissions were nonelective (85% of total).

### 3.2. Hemorrhagic Complications

**Figure 1:**
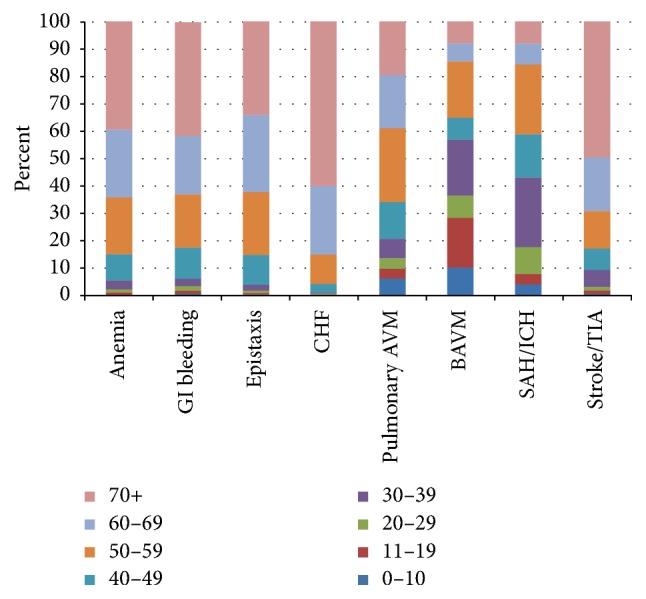
Age and frequency of specific complications. GI = gastrointestinal; CHF = congestive heart failure; AVM = arteriovenous malformation; BAVM = brain arteriovenous malformation; SAH = subarachnoid hemorrhage; ICH = intracranial hemorrhage; TIA = transient ischemic event.

**Figure 2:**
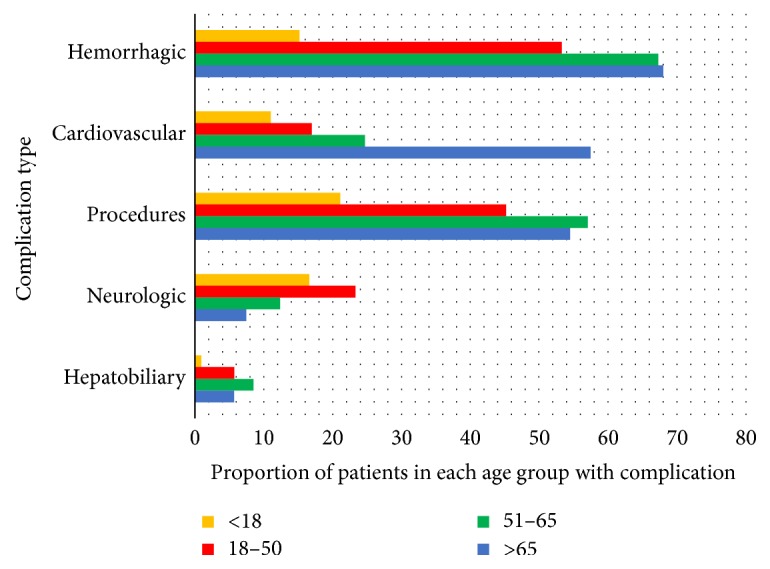
Proportion of patients with a specific complication in each age group.

**Figure 3:**
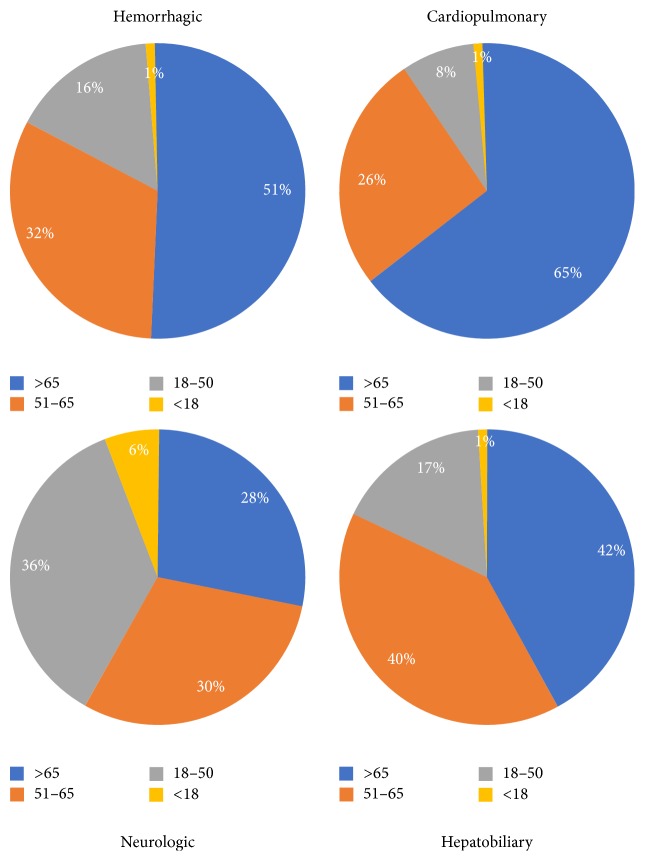
Prevalence of complications by age group.

Complications and procedures in hospitalized HHT patients are shown in Table 2 and Figures 1, 2, and 3. Hemorrhagic complications were the most frequent type of complication and occurred in 62.7%  (*n* = 6455) of HHT-related hospitalizations. Anemia was the most frequent hemorrhagic complication accounting for 85%  (*n* = 5484) of total bleeding events. Epistaxis accounted for 25.9%  (*n* = 1669) of events and GI bleeding for 17.2%  (*n* = 1111) of total hemorrhagic complications. Age was significantly associated with the prevalence of bleeding related complications as 15.3%, 53.3%, 67.3%, and 68.1% of the <18, 18–50, 51–65, and >65 age groups were hospitalized with bleeding complications, respectively (*P* < .0001). Patients older than 50 accounted for a total of 82.9%  (*n* = 5353) of all bleeding related complications.

### 3.3. Cardiopulmonary Complications

Cardiopulmonary complications were the second most common group of complications noted in the HHT population affecting 41.0%  (*n* = 4216) of patients. Congestive heart failure was frequent and accounted for 48.7%  (*n* = 2052) of total cardiopulmonary complications. Coronary artery disease (40.7%, *n* = 1718), atrial fibrillation (33.8%, *n* = 1423), and pulmonary hypertension (22.6%, *n* = 950) being the other common cardiopulmonary complications noted. Overall, there was a significant age-related increase in cardiopulmonary complications with 11.1% 17.0%, 34.7%, and 57.5% of patients in the <18, 18–50, 51–65, and >65 age groups suffering from cardiopulmonary complications, respectively (*P* < .0001). Overall, 90.1% (3836) of cardiopulmonary complications occurred in those older than 50 years of age. Pulmonary AVMs were noted in 1% of cases with the highest number noted in the 18–50 age group. Overall, congestive heart failure was the 2nd most frequent complication after anemia in HHT patients.

### 3.4. Neurological Complications

Neurological events were the third most common category of complications seen in 12.4%  (*n* = 1276) of all admissions. Seizures were the most common neurological complication seen in 628 cases (49.2% of total neurological events) followed by headache (28.4%, *n* = 363), stroke (18.4%, *n* = 235), and brain AVMs (6%, *n* = 88). The highest rates of neurological complications were noted in the 18–50 age group (23.4%, *n* = 453) followed by the <18 age group (16.8%, *n* = 77), the 51–65 age group (12.4%, *n* = 385), and the >65 age group (7.5%, *n* = 361).

### 3.5. Hepatic Complications

Hepatic complications occurred in 6.4%  (*n* = 660) of all hospitalizations. Cirrhosis accounted for 67.9%  (*n* = 448) of total hepatobiliary complications followed by biliary complications (25.6%, *n* = 169) and portal hypertension (20.2%, *n* = 133). Hepatic complications were most common in the 51–65 age group (*n* = 265, 8.6%) and least common in the <18 age group (*n* = 5, 1.1%). Patients older than 50 accounted for 82%  (*n* = 542) of total hepatobiliary complications.

### 3.6. Procedures

Procedures were noted in a total of 5357 admissions (52.1%). Blood transfusions were the most frequent accounting for 73.0%  (*n* = 3908) of all procedures. This was followed by upper endoscopy (18.3%, *n* = 981), lower endoscopy (8.5%, *n* = 456), surgical ligation for epistaxis (7.5%, *n* = 404), nasal packing (7.0%, *n* = 375), and endovascular treatment of epistaxis (2.8%, *n* = 151). Age was associated with a significant increase in procedural volume with 21.2%, 45.2%, 57.9%, and 54.5% of patients in the <18, 18–50, 51–65, and >65 age groups undergoing procedures, respectively. Overall, 83%  (*n* = 5594) of procedures were performed in those older than 50 years of age.

### 3.7. In Hospital Outcomes

In hospital outcomes are summarized in Table 3. The overall in-hospital mortality rate was 1.9% with a significant increase noted with age (*P* < .0001). Rates of discharge to home were the highest in the <18 age group and, concurrently, the rates of discharge to long term rehabilitation facilities were the highest in the >65 age group (*P* < .0001 for both).

## 4. Discussion

Our data provide the first comprehensive assessment of the impact of age on various complications in a very large nationwide sample of hospitalized HHT patients. We show for the first time that the vast majority (>75%) of hospitalizations occur in HHT patients older than 50 years of age. In addition, more than 80% of bleeding and cardiopulmonary and hepatobiliary complications occurred in this same age group. Although the primary reason for hospitalization could not be ascertained from this database, the progressive increase in the rates of bleeding and cardiovascular and hepatobiliary complications suggest that these were the likely drivers for hospitalization in the older age groups.

### 4.1. Age and HHT-Related Complications

Several previous studies have investigated the effect of age on the prevalence and complication rates amongst HHT patients [2, 4, 6, 10, 11]. These studies appear to show a definite age-related HHT penetrance. For example, Plauchu showed that age was associated with increasing rates of bleeding complications amongst HHT patients [2]. However many of these studies were limited by their small sample size and assessment of a community based rather than a hospitalized sample of HHT patients. This is the first study that incorporates a very large number (*n* = 10293) of hospitalized HHT patients across the United States. The study sample represents the entire age and demographic, socioeconomic, and geographic spectrum in the United States allowing for several important and specific observations to be made with regard to organ specific complications.

We have previously shown a high rate of bleeding related complications amongst hospitalized HHT patients [7]. The present study confirms a very strong trend towards higher rates of these bleeding related complications in older patients. The fact that over 80% of bleeding related complications occurred in HHT patients older than 50 is both astonishing and sobering. Our data appears to be internally consistent given that the rates of hemorrhagic complications correlates very well with increasing rates of blood transfusion, upper and lower GI endoscopies, and surgical/endovascular epistaxis treatment procedures in the older age groups. These findings should compel us to adopt a more systematic approach towards the screening and management of anemia, epistaxis, and GI bleeding in HHT patients. Earlier diagnoses and close follow-up at HHT centers of excellence will likely result in improved outcomes [12]. The lack of FDA (Food and Drug Administration, USA) approved therapies for HHT-related bleeding limits therapeutic options but strongly advocates for the urgent study of Bevacizumab and other antiangiogenic agents for these complications [13]

A very similar age trend is also noted in cardiovascular and hepatobiliary complications with more than 90% of cardiopulmonary and 82% of hepatobiliary complications occurring in those older than 50. Congestive heart failure alone accounted for 30% of all cardiopulmonary complications and pulmonary hypertension was noted in 14% of cases. Although the exact etiology of these complications (HHT- versus non-HHT-related) can be debated, the age-related increases in anemia, CHF, atrial fibrillation, pulmonary hypertension, and hepatobiliary complications all appear to point towards worsening AVM related disease burden in older HHT patients. This is the first study to show these surprisingly high rates of cardiopulmonary and hepatobiliary complications amongst hospitalized HHT patients. Current HHT treatment guidelines do not advocate screening for liver involvement in asymptomatic HHT patients [14]. These recommendations may need to be revised given the significant rates of hepatobiliary complications noted in this study. In addition, one must also remember that these patients can remain free of clinical and laboratory abnormalities even with advanced liver AVM involvement. Thus, the lack of a systematic screening program for liver involvement (or high output heart failure) may delay initiation of potentially beneficial antiangiogenic therapy [13] resulting in increased morbidity and mortality. One can argue for the benefits of a screening echocardiogram at age 50 (specifically looking for high output heart failure) followed by repeated screening at 3–5 yearly intervals in all patients with evidence for liver involvement.

Neurological complications were somewhat unique in that the highest incidence of seizures, headaches, brain abscess, ICH, SAH, cerebral aneurysm, and spinal vascular malformations occurred in the 18–50 age group. This is a very interesting and somewhat counterintuitive observation, given that de novo CNS vascular malformations rarely (if at all) occur in adult HHT patients. There are several potential explanations for this significant time-delay in the occurrence of neurological complications: (1) poor knowledge and understanding of HHT amongst patients and healthcare providers resulting in prolonged diagnostic delays into adulthood [15], (2) sporadic and nonstandardized screening protocols for PAVMs, and (3) a general underappreciation for the risk that asymptomatic and otherwise “small” PAVMs pose for embolic CNS disease in younger patients. Our data convincingly dispels the notion that CNS complications predominantly occur in the pediatric age group with a lower risk in older patients. Current guidelines do recommend screening for CNS AVMs in pediatric patients (preferably at the time of initial HHT diagnosis). Recommendations for PAVM screening pulmonary involvement at the time of diagnosis and before the age of 18 should be further emphasized [14].

### 4.2. Limitations and Strengths of the Study

Limitations of our study include potential coding and documentation errors which remain a concern for any data obtained from large administrative databases. Additionally, the lack of individual patient level clinical and genetic data prevents the assessment of individual HHT characteristics and the number of Curacao criteria met. These factors have been previously shown to influence rates of complications and outcomes [5, 16–18]. Moreover, outcomes are limited to the in-hospital period with no follow-up data available post hospitalization. It is also not possible to determine whether patients were primarily admitted for a HHT-related complication or whether HHT was a secondary (and possibly inactive) problem at the time of hospitalization. Strengths of this study include the large number of HHT patients obtained from a representative nationwide sample. The NIS database is probably one of the few sources from which a large nationwide sample of hospitalized HHT patients can be obtained including patients from different geographic and ethnic backgrounds, rural versus urban settings, high versus low socioeconomic status, and those treated at small rural community hospitals versus urban tertiary referral centers. Thus, this study likely provides a “real world” picture of hospitalized HHT patients across the US.

## 5. Conclusion

In a large nationwide sample of hospitalized HHT patients in the USA, the vast majority of serious complications occurred in those older than 50 years of age. There was also a significant, age-related increase in the rates of hepatobiliary, cardiovascular, and bleeding related complications amongst these patients. These findings provide convincing evidence that older patients bear a vast majority of the HHT-related disease burden especially in relation to bleeding and cardiovascular complications.

## Figures and Tables

**Table 1:** Baseline characteristics in different age groups.

Age	<18	18–50	51–65	≥65	*P* value
*N*	458	1938	3097	4800	
Elective admission	70 (16.9)	353 (20.5)	483 (17.6)	629 (15.8)	<.0001
*N* (%) Female	254 (55.7)	1247 (64.3)	1732 (55.9)	2969 (61.9)	<.0001
Race					
White	251 (65.0)	1121 (70.5)	1811 (71.0)	3223 (83.3)	<.0001
Black	39 (10.1)	249 (15.7)	381 (14.9)	288 (7.4)	
Hispanic	63 (16.3)	163 (10.3)	286 (11.2)	256 (6.6)	
Asian	5 (1.3)	16 (1.0)	33 (1.3)	23 (0.6)	
Income quartile					
1	63 (13.8)	444 (23.5)	780 (25.9)	913 (19.3)	<.0001
2	107 (23.5)	434 (23.0)	735 (24.4)	1190 (25.2)	
3	144 (31.6)	487 (25.8)	716 (23.8)	1243 (26.3)	
4	142 (31.1)	524 (27.7)	778 (25.9)	1379 (29.2)	
CCI	0.0 (0.2)	0.5 (0.9)	0.9 (1.2)	1.2 (1.2)	<.0001

CCI = Charlson comorbidity index.

**Table 2:** Complication rates and procedures in different age groups.

	Age groups				
Complication	<18	18–50	51–65	>65	*P* value
*N* (%)	458	1938	3097	4800	
*Hemorrhagic*	70 (15.3)	1032 (53.3)	2085 (67.3)	3268 (68.1)	<.0001
Anemia	53 (11.6)	867 (44.7)	1789 (57.8)	2775 (57.8)	<.0001
Epistaxis	11 (2.4)	265 (13.7)	592 (19.1)	801 (16.7)	<.0001
GI bleed	19 (4.2)	192 (9.9)	328 (10.6)	572 (11.9)	<.0001
Hematemesis	3 (0.7)	26 (1.3)	36 (1.2)	43 (0.9)	.29
Hemoptysis	3 (0.7)	4 (0.2)	12 (0.4)	31 (0.7)	.09
*Cardiovascular*	51 (11.1)	329 (17.0)	1075 (24.7)	2761 (57.5)	<.0001
CHF	3 (0.7)	94 (4.9)	467 (15.1)	1488 (31.0)	<.0001
Coronary artery disease	0 (0.0)	81 (4.2)	371 (12.0)	1266 (26.4)	<.0001
Atrial fibrillation	0 (0.0)	31 (1.6)	298 (9.6)	1094 (22.8)	<.0001
Pulmonary hypertension	44 (9.6)	161 (8.3)	295 (9.5)	450 (9.4)	.48
MI	0 (0.0)	11 (0.6)	52 (1.7)	108 (2.3)	<.0001
Pulmonary embolism	0 (0.0)	25 (1.3)	43 (1.4)	49 (1.0)	.046
DVT	0 (0.0)	16 (0.8)	42 (1.4)	50 (1.0)	.04
Pulmonary AVM	8 (1.8)	20 (1.0)	33 (1.1)	21 (0.4)	.0006
Vfib/cardiac arrest	0 (0.0)	3 (0.2)	12 (0.4)	17 (0.4)	.28
*Neurologic*	77 (16.8)	453 (23.4)	385 (12.4)	361 (7.5)	<.0001
Seizure	38 (8.3)	211 (10.9)	207 (6.7)	172 (3.5)	<.0001
Headache	25 (5.5)	186 (9.6)	99 (3.2)	53 (1.1)	<.0001
Stroke	2 (0.4)	39 (2.0)	56 (1.8)	138 (2.9)	.0004
BAVM	20 (4.4)	37 (1.9)	22 (0.7)	9 (0.2)	<.0001
TIA	0 (0.0)	9 (0.5)	20 (0.7)	40 (0.8)	.09
Cerebral abscess	0 (0.0)	23 (1.2)	24 (0.8)	8 (0.2)	<.0001
ICH	1 (0.2)	16 (0.8)	8 (0.3)	6 (0.1)	<.0001
SAH	2 (0.4)	12 (0.6)	7 (0.2)	0 (0.0)	<.0001
Cerebral aneurysm	1 (0.2)	10 (0.5)	9 (0.3)	11 (0.2)	.27
Spinal vascular malformation	1 (0.2)	4 (0.2)	0 (0.0)	0 (0.0)	.0009
*Hepatobiliary complications*	5 (1.1)	113 (5.8)	265 (8.6)	277 (5.8)	<.0001
Cirrhosis	2 (0.4)	67 (3.5)	201 (6.5)	178 (3.7)	<.0001
Biliary complications	2 (0.4)	32 (1.7)	54 (1.7)	81 (1.7)	.22
Portal hypertension	1 (0.2)	20 (1.0)	61 (2.0)	51 (1.1)	.0004
Chronic liver failure	1 (0.2)	10 (0.5)	16 (0.5)	19 (0.4)	.71
Jaundice	0 (0.0)	4 (0.2)	9 (0.3)	7 (0.2)	.39
Acute liver failure	0 (0.0)	5 (0.3)	1 (0.0)	6 (0.1)	.12
*Procedures*	97 (21.2)	875 (45.2)	1768 (57.1)	2617 (54.5)	<.0001
Transfusion	59 (12.9)	586 (30.2)	1343 (43.4)	1920 (40.0)	<.0001
Upper endoscopy	15 (3.3)	184 (9.5)	286 (9.2)	496 (10.3)	<.0001
Lower endoscopy	8 (1.8)	80 (4.1)	124 (4.0)	244 (5.1)	.002
Surgical ligation for epistaxis	5 (1.1)	62 (3.2)	141 (4.6)	196 (4.1)	.001
Nasal packing	2 (0.4)	31 (1.6)	102 (3.3)	240 (5.0)	<.0001
Endovascular treatment of epistaxis	16 (3.5)	48 (2.5)	51 (1.7)	36 (0.8)	<.0001
Cardiac catheterization	2 (0.4)	40 (2.1)	95 (3.1)	138 (2.9)	.003
Hemodialysis	0 (0.0)	35 (1.8)	30 (1.0)	67 (1.4)	.005
IVC filter	0 (0.0)	19 (1.0)	34 (1.1)	39 (0.8)	.11
CPR	1 (0.2)	7 (0.4)	5 (0.2)	7 (0.2)	.31

GI = gastrointestinal; CHF = congestive heart failure; MI = myocardial infarction; DVT = deep vein thrombosis; AVM = arteriovenous malformation; Vfib = ventricular fibrillation; BAVM = brain arteriovenous malformation; TIA = transient ischemic attack; ICH = intracranial hemorrhage; SAH = subarachnoid hemorrhage; IVC = inferior vena cava; CPR = cardiopulmonary resuscitation.

**Table 3:** Outcomes in different age groups.

Age	<18	18–50	51–65	≥65	*P* value
*N*	458	1938	3097	4800	
Long term	5 (1.1)	64 (3.3)	220 (7.1)	919 (19.2)	<.0001
Home	417 (91.1)	1663 (85.9)	2431 (78.5)	3011 (62.8)	<.0001
Short	10 (2.2)	65 (3.4)	80 (2.6)	105 (2.2)	.048
Death	3 (0.7)	21 (1.1)	44 (1.4)	132 (2.8)	<.0001
LOS	4.1 (6.0)	4.3 (5.6)	4.4 (5.5)	4.8 (5.8)	.0006
Charge	29908 (71669)	32793 (60523)	30616 (48943)	28149 (46686)	.008
Iatrogenic complication	33 (7.2)	119 (6.1)	214 (6.9)	280 (5.8)	.22

Long-term = discharged to a long term care facility; home = discharged to home; short = discharged to a short term rehabilitation facility; LOS = hospital length of stay; charge = total hospital charges.
